# The Book World of Medicine and Science

**Published:** 1894-09-29

**Authors:** 


### The Book World of Medicine and Science.

Disease and Race. By Jadroo. (London: Swan Sonnenschein and Co., Paternoster Square.)

In this little book is broached a theory, bold to the point of audacity, of universal inoculation, not of the Pasteurian, but of the natural and unconscious sort, carried on through countless generations. That is, that when a race has suffered long from a disease it attains complete, or at least comparative, immunity. To this, aided by vaccination, we owe the marked decrease of small-pox. The disease, even when it does attack an Englishman, very rarely leads to a virulent epidemic, though, says "Jadroo," "Let the same infection be placed in the midst of some African tribe on, say, the banks of the Albert Nyanza, and see if it would not give an extremely good account of itself." Measles, with us a childish ailment to which no one gives much heed, is virulent and fatal among the Fiji Islanders. That this cannot be explained by the mere weakness of an "inferior" race, for these races show themselves comparatively immune to yellow fever, malaria, and such diseases, though these dig the white man's grave. The inference is that in the different races repeated infection has brought about immunity, as in the individual one attack of certain diseases precludes the possibility of another. The evidence which "Jadroo" brings in support of his theory is strong, but not wholly convincing; but there exists (and if he knows of it we are surprised that he did not quote it) the testimony of Humboldt that European sailors, themselves perfectly healthy, convey European diseases to the South Sea Islanders. "Fevers, cholera, and other epidemics have frequently committed such devastations among the inhabitants of these islands that the natives were unable to bury their dead, while the sailors who imported them were perfectly healthy." The inference is that, with the sailors, the disease had reached what "Jadroo" calls the "saturation point," when it fails to have any effect, though it can still be transmitted in full virulence to a fresh soil.

To this racial saturation, and the immunity it confers, is attributed the disappearance of leprosy and other diseases recorded as having once been endemic in Europe, with the aid of another theory which "Jadroo" has evolved, viz., that two diseases meeting in the same organism produce a third, a hybrid disease, repeating, though in probably a milder form, the characteristics of both parents. Thus a new disease is formed, and the two elder ones die out. Syphilis "Jadroo" regards as the offspring of leprosy and gonorrhœa, though we confess that, as syphilis is generally admitted to have been brought back by the companions of Columbus from the West Indies, we do not see that the ancestry is clearly established, but our author insists that it existed long before, and was regarded as a form of leprosy. Small-pox comes from the union of measles and plague, typhoid from the mating of typhus and malarial fever, while diphtheria is the child of scarlet fever and some unnamed putrefactive organism. We confess to thinking this a somewhat fanciful, though by no means impossible hypothesis, which would require handling on a larger scale than it can receive in this slim volume. The book is clever and original; it is also written in a bright and readable style; but we doubt if "Jadroo" has, in this offhand fashion, settled the problem of disease and race. But we should be glad to have a fuller statement of his ingenious hypothesis.

